# Editorial: Vertigo, tinnitus, and cognition

**DOI:** 10.3389/fnins.2024.1399340

**Published:** 2024-05-08

**Authors:** Xingxing Lu, Haijin Yi

**Affiliations:** Department of Otolaryngology, Head and Neck Surgery, Beijing Tsinghua Changgung Hospital, School of Clinical Medicine, Tsinghua University, Beijing, China

**Keywords:** vestibular system, vestibular dysfunction, cognitive function, dizziness, tinnitus

The vestibular system plays a role in maintaining the body's orientation and balance. A growing number of studies have demonstrated that vestibular dysfunction can affect various aspects of cognitive function, including executive function, memory, attention, psycho-psychological cognition, and visuospatial ability (Liu et al., 2019). Of particular note is the correlation between vestibular impairment, navigation, and spatial memory. In clinical practice, vestibular dysfunction is mainly manifested by dizziness/dizziness. Tinnitus is a common auditory symptom that involves the conscious perception of sound without any corresponding auditory stimulation. Evidence suggests that tinnitus patients have difficulties with cognitive functions, such as attention and memory. However, we need a better understanding of the physiological mechanisms underlying how tinnitus and dizziness affect cognitive function in order to predict whether controlling specific vestibular symptoms or tinnitus will improve cognitive function.

Yang et al. investigated the potential correlation between tinnitus and the risk of various cognitive impairments, such as dementia, impaired learning attention, anxiety, depression, and insomnia. They discovered that there may be a higher chance of cognitive deficits with tinnitus. Furthermore, individuals over the age of 60 exhibit a greater vulnerability to cognitive impairment than younger patients. These authors' research provided important evidence to support the hypothesis that tinnitus and cognitive decline are related.

Furthermore, the study by Zhang et al. explored changes in brain function in tinnitus patients at the electrophysiological level. This work explored the aberrant dynamics of electroencephalogram (EEG) microstates and their correlations with tinnitus features in patients with vestibular schwannoma (VS). Their EEG microstate analysis revealed that VS patients with tinnitus exhibited an increased frequency of microstate C compared to those without tinnitus. Additionally, correlation analysis showed that the Tinnitus Handicap Inventory score was positively correlated with microstate C frequency and negatively correlated with microstate A duration. These results imply that there are notable differences in the temporal and syntactic features of EEG microstates between VS patients with and without tinnitus, which may be indicative of aberrant neural resource allocation and functional brain activity transition. Their findings offer a theoretical framework for the creation of a variety of tinnitus treatments.

Research has shown that chronic dizziness, such as vestibular migraine, is more prone to cognitive impairment. Zhe et al. investigated the resting-state functional connectivity (FC) patterns during the interictal period in vestibular migraine (VM) by combining data-driven voxel-wise degree centrality (DC) calculation and seed-based FC analyses. According to their findings, there are specific whole-brain FC anomalies in VM patients. Patients with VM displayed lower DC values in the medial prefrontal cortex and higher DC values in the right occipital lobe. The study shed more light on the complexity of the mechanisms underlying VM. These FC and DC deficits were negatively correlated with the clinical data of the patients, such as episode frequency, DHI score, and pain severity, indicating that lower FC is significantly associated with the impact of recurrent migraine episodes on day-to-day functioning.

Studies have started to point to a connection between vestibular dysfunction and cognitive impairment. It is not yet known whether medication can reverse cognitive decline. The Montreal Cognitive Assessment and the Dizziness Handicap Inventory were used in the study by Zhong et al. to evaluate the cognitive function, vertigo symptoms, and associated physical, functional, and emotional effects of the MD patients over the course of 3, 6, and 12 months following treatment. According to the authors, this cognitive impairment may improve with successful treatment. The idea that vestibular dysfunction is a potentially controllable risk factor for cognitive decline is supported by their findings.

The review by Smith et al. summarized key literature and recent updates related to vestibular cognitive dysfunction and pointed to a significant degree of redundancy in the central vestibular pathways, which is supported by the amazing ability of patients to compensate for deficiencies in their peripheral vestibular systems and, with the right care, recover from or manage the majority of vestibular disorders. Combined with Zhong et al. research, it is possible to further improve cognition by improving vestibular dysfunction. The authors' article also provides recommendations and priority areas for healthcare professionals who assess and treat vestibular disorders, and for researchers developing cognitive models and rehabilitation interventions.

These works on the relationship and mechanism of action between dizziness, tinnitus, and cognition provide a basis for potential interventions to improve partial cognitive function through the treatment of tinnitus, vestibular stimulation, and vestibular rehabilitation, providing new treatment ideas and methods for the improvement of cognitive function.

## Author contributions

HY: Writing – review & editing. XL: Writing – original draft.
